# An atlas of bovine gene expression reveals novel distinctive tissue characteristics and evidence for improving genome annotation

**DOI:** 10.1186/gb-2010-11-10-r102

**Published:** 2010-10-20

**Authors:** Gregory P Harhay, Timothy PL Smith, Leeson J Alexander, Christian D Haudenschild, John W Keele, Lakshmi K Matukumalli, Steven G Schroeder, Curtis P Van Tassell, Cathy R Gresham, Susan M Bridges, Shane C Burgess, Tad S Sonstegard

**Affiliations:** 1 USDA-ARS US Meat Animal Research Center, State Spur 18 D, Clay Center, NE 68901, USA; 2 USDA-ARS Fort Keogh Livestock and Range Research Laboratory, 243 Fort Keogh Road, Miles City, MT 59301, USA; 3 Illumina, Inc., 25861 Industrial Boulevard, Hayward, CA 94545, USA; 4 Department of Bioinformatics and Computational Biology, George Mason University, 10900 University Blvd, Manassas, VA 20110, USA; 5 USDA-ARS Bovine Functional Genomics Laboratory, 10300 Baltimore Avenue, Bldg 200 Rm 2A BARC-East, Beltsville, MD 20705, USA; 6 Department of Computer Science, Mississippi State University, Mississippi State, MS 39762, USA; 7 Department of Basic Sciences, College of Veterinary Medicine, Mississippi State University, Mississippi State, MS 39762, USA

## Abstract

**Background:**

A comprehensive transcriptome survey, or gene atlas, provides information essential for a complete understanding of the genomic biology of an organism. We present an atlas of RNA abundance for 92 adult, juvenile and fetal cattle tissues and three cattle cell lines.

**Results:**

The Bovine Gene Atlas was generated from 7.2 million unique digital gene expression tag sequences (300.2 million total raw tag sequences), from which 1.59 million unique tag sequences were identified that mapped to the draft bovine genome accounting for 85% of the total raw tag abundance. Filtering these tags yielded 87,764 unique tag sequences that unambiguously mapped to 16,517 annotated protein-coding loci in the draft genome accounting for 45% of the total raw tag abundance. Clustering of tissues based on tag abundance profiles generally confirmed ontology classification based on anatomy. There were 5,429 constitutively expressed loci and 3,445 constitutively expressed unique tag sequences mapping outside annotated gene boundaries that represent a resource for enhancing current gene models. Physical measures such as inferred transcript length or antisense tag abundance identified tissues with atypical transcriptional tag profiles. We report for the first time the tissue-specific variation in the proportion of mitochondrial transcriptional tag abundance.

**Conclusions:**

The Bovine Gene Atlas is the deepest and broadest transcriptome survey of any livestock genome to date. Commonalities and variation in sense and antisense transcript tag profiles identified in different tissues facilitate the examination of the relationship between gene expression, tissue, and gene function.

## Background

Comprehensive surveys of transcript abundance among tissues, often referred to as gene atlases, are relatively few [1-10], but provide novel and detailed insights into the genomic biology of the organism surveyed. For example, genomic studies often reveal chromosomal segments harboring variation affecting a trait, and knowledge of the expression profiles of genes lying in these segments enhances selection of candidate genes for further investigation. From another perspective, knowledge of the tissues in which a particular transcript is expressed may provide additional evidence about gene function. The utility and quality of a gene atlas for these types of analyses is limited by its depth (defined as the sensitivity to rare transcripts relative to abundant transcripts) and breadth, represented by the diversity of the tissue types and developmental stages.

The emergence of next generation sequencing (NGS) technologies has expanded the depth available for creation of gene atlases by providing an alternative to DNA microarray approaches for monitoring gene expression [1]. Profiling using NGS has a greater capacity to represent all extant transcripts (since microarrays monitor only those sequences for which probes have been or can be created) and wider dynamic range (up to the limit of the efficiency of cDNA synthesis, depending on number of sequences collected). Two approaches to enumerate transcripts with NGS have been developed, either based on sequencing specific tags related to restriction sites in the cDNA (digital gene expression (DGE)) or random cDNA fragments (RNAseq) [2]. The former approach was the only one available making use of NGS at the time of this transcriptome study based on restriction digestion of bovine cDNA with the enzyme DpnII and capture of 20-base tags (including the GATC restriction site) from the 3'-most restriction site. The disadvantage of DGE is that it fails to capture expression information from transcripts lacking DpnII sites (approximately 3% of current bovine gene models do not have predicted DpnII recognition sequences). On the other hand, collapsing tag counts to a unique locus to precisely quantify transcript abundance using DGE tags can be more straightforward than the assembly of short, sometimes non-overlapping reads, especially for organisms lacking high quality genome sequences and annotation (as is the case for cattle).

The breadth of existing gene atlases varies, with some aiming for extreme breadth in a limited set of tissue types, such as the adult mouse brain atlas with a fine-grained localization of expression [4], and others being less specialized, such as the mouse atlas describing approximately 34 tissue types at multiple developmental stages [5]. In cattle, where the bulk of research is focused on tissues important to efficient production of unadulterated beef, additional considerations in selecting tissues for a gene atlas come into play. For example, cattle research is more concerned with variation in gene expression among muscle classes, fat depots, or the digestive system than is normally the case in mouse studies. In contrast, much mouse research is related to basic studies in developmental biology as a model organism and, thus, a useful gene atlas for mice will tend to concentrate more on breadth across developmental stages than breadth across subclasses of tissue types within a stage (such as different muscles). The breadth of an atlas can be evaluated in the light of tissue ontologies, such as that in the Braunschweig Enzyme Database (BRENDA) classification system [6,7].

In general, there are impediments to drawing biological inferences from transcriptional profiles. These barriers include the complexity of biological systems, the lack of knowledge about the details of cattle-specific biological processes, and the fact that the cattle draft genome is relatively new and not as well annotated as more mature genomes such as human or mouse. The Bovine Gene Atlas (BGA) was created to address some of these shortcomings. For instance, associating Bovidae-specific tissues, such as the rumen, with other tissues with a similar transcript profile that are also present and well studied in other non-ruminant organisms will be a useful first step to seed investigations of biological processes specific to Ruminantia species. We collected a total of 95 samples (including three cell lines) spanning one fetal stage, one juvenile stage, and a number of adult animals, and constructed the first BGA, which to our knowledge is also the first organism-wide atlas to be constructed using NGS technology. The BGA is available for viewing online within a genomic context [8].

## Results and discussion

### Breadth and depth of the BGA

**Figure 1:**
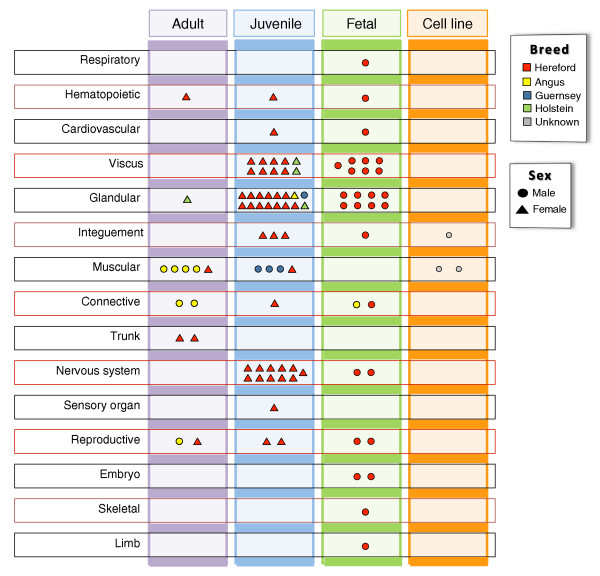
**The 95 samples comprising the Bovine Gene Atlas**. The samples are classified according to BRENDA tissue class, developmental stage, breed, and sex. Most tissues were sampled from animals related to L1 Dominette, the Hereford cow whose genome was sequenced.

The breadth of the tissues in the BGA is illustrated in Figure 1. The majority of the tissues were harvested from animals related to L1 Dominette 01449, the Hereford cow whose genome was sequenced [9,10]. Most of these samples were from her male late-gestation fetus and juvenile daughter to reduce the impact of polymorphisms on analyses and capture changes in the transcriptomes early in the life cycle that may influence the adult state. The tissues selected were chosen based on their presumed influence on livestock traits, most of which are growth related. Therefore, the atlas consists in large part (58%) of endocrine (BRENDA [6,7] gland), alimentary (BRENDA viscus), and nervous tissues that provide for a wide diversity in expression profiles. In addition, muscle and fat depots from adult and juvenile steers were sampled to compare transcript levels among these economically important tissues. A complete list of specific tissues can be found in Additional file 1.

**Figure 2:**
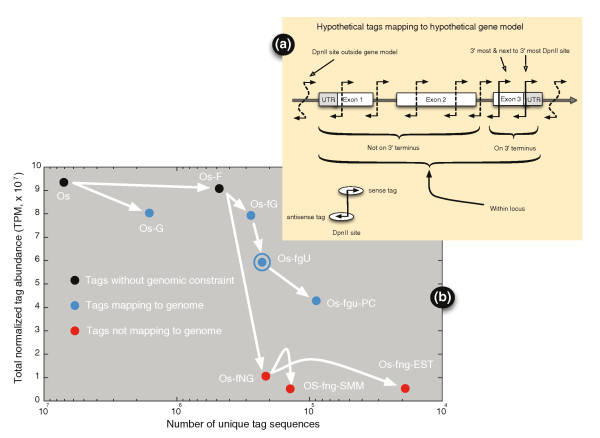
**Tag processing**. **(a) **Tags mapping to a hypothetical gene model, definition of terms. Sense tags were defined to be those tags on the same strand as the gene model, antisense tags were on the opposite strand. The 'On 3' terminus' tags were defined to be on the 3' terminus derived from the two downstream-most positions on the transcript, while the rest of the tags within the gene boundaries were defined to be 'Not on 3' terminus'. The union of these two sets was defined as tags 'Within locus'. **(b) **Tag genome mapping and filtering. The ordinate 'Total normalized tag abundance' is the sum of all normalized tag counts (TPM) over all tissues, while the abscissa 'Number of unique tag sequences' is the set of tags from all 95 tissues. Os, operative set of all observed tags that do not possess an ambiguous base; Os-G, subset of Os tags perfectly mapping to the draft bovine genome; Os-F, subset of Os tags found in at least ten tissues and/or have a tag abundance of 2 TPM or greater in at least one tissue; Os-fG, subset of Os-F tags that mapped to the draft bovine genome; Os-fgU, subset of Os-fG tags with unique matches to the draft bovine genome - the Os-fgU tag set is analyzed further in Additional file 1 and is marked with a concentric circle; OS-fgu-PC, the subset of Os-fgU tags mapping to protein-coding genes; OS-fNG, the subset of Os-F tags that do not map to the draft bovine genome; OS-fng-SMM, the subset of Os-fNG tags that map back to the draft genome because of a single base mismatch at tag base positions 5 to 20; Os-fng-EST, subset of the Os-fNG tags that map to bovine ESTs.

The depth of the BGA is demonstrated with the observation of 300,268,171 tags representing 7,296,656 unique 20-base sequences collected from 92 tissues and three cell lines for a total of 94,997,401 tags per million (TPM). TPM is a normalized measure of tag count, where each library was normalized to contain 1 million TPM. The slight deviation (0.003%) of the observed tag count from the theoretical 95 × 10^6 ^was due to rounding errors. Eliminating tags with indeterminate bases (N) and adaptor sequence yielded 296,179,417 tags consisting of 7,280,319 unique 20-base sequences for a total of 93,750,421 TPM. This set was defined to be the operative set (Os) of all completely defined tags from which mapping and filtering can be performed, as illustrated in Figure 2b. First, Figure 2a illustrates terminology used in describing the way in which tags may map to the draft genome and the gene models annotated on the bovine draft genome sequence [9]. Out of 24,294 bovine RefSeq transcripts [11], 23,481 (96.7%) had a DpnII site that could potentially contribute to this atlas. Many transcripts contain multiple predicted restriction sites, and some transcripts may contain sites not annotated as a result of polymorphisms between animals. The use of the index cow for sequencing and her immediate offspring should minimize such occurrences. Annotation of the RefSeq set on the draft genome sequence can be used to classify the tags according to their relative location: either within or outside annotated gene boundaries, in exons or introns within a gene boundary, or in the UTRs of the transcript. Furthermore, the tags may match the sense or antisense strand of the genomic DNA relative to the gene model, and may either match the 3'-most predicted DpnII site as intended in the protocol, or one of the upstream sites (if present) depending on a number of factors, such as alternative splice forms or incomplete DpnII digestion. Considering only the two 3'-most DpnII sites, the primary 3'-most DpnII site is associated with 91.5% of the observed tag abundance, while the next to 3'-most DpnII site constituted 8.5% of the observed tag abundance, suggesting that the protocol is yielding acceptable results.

Figure 2b describes the results of mapping tags to the draft genome, starting with the Os where 1,588,191 distinct tag sequences (Os-G) aligned perfectly to the draft genome for a total tag abundance of 80,326,698 TPM. In other words, only 21.8% of the Os unique tag sequences mapped to the draft genome, but these tags represented 85.7% of the Os tag abundance. This was due mainly to a diverse set of singleton tags that may represent sequence errors (or other phenomena; see section on non-matching tags below). To more efficiently remove artifactual tags, an additional criterion was used to eliminate tags with very low abundance (less than 2 TPM). However, because tags were collected from a relatively larger number of tissues compared to other transcriptomic investigations, transcripts from lowly transcribed genes, present at levels below 2 TPM, were included for consideration if they were present in at least ten libraries on the grounds that their presence in at least ten libraries suggests that the tag sequences were not the result of sequencing error. This 2 TPM/ten tissue constraint was applied to subsequent analyses in this report, and resulted in 483,788 unique 20-base tag sequences (Os-F) totaling 89,858,285 TPM among all 95 samples, of which 272,610 unique tag sequences (Os-fG; 56.3% of the Os-F) amounted to 79,282,121 TPM (88.2% of the Os-F) mapped to the draft genome. This 2 TPM/ten tissue filter reveals that only 6.65% of the unique tag sequences account for 95.8% of the total normalized Os tag abundance. Thus, requiring the tags to map to a single position in the draft genome reduces the Os-fG tag set to 227,481 unique tag sequences (Os-fgU; 83.4% of the Os-fG) for a total tag abundance of 59,373,362 TPM (74.9% of the Os-fG) in all samples, and accounting for 66.1% of the observed total tag abundance in the Os-F. This requirement results in a floor in the estimate in the number of unique transcript sequences and genes observed in all tissues. The constraint that the tags must map to a single position in the draft genome has been applied to subsequent analyses in this report (that is, the Os-fgU subset of tags was used for all subsequent analyses) since tags that do not map uniquely to the draft genome cannot be unambiguously assigned to particular loci. For instance, the subset of tags that mapped within the gene boundaries of annotated protein-coding loci yielded 87,764 unique tag sequences (Os-fgu-PC) mapping to 16,517 loci with distinct GeneIDs, totaling 42,681,813 TPM.

Using a filter that required tags to match a single location in the draft genome was instituted because this simplified the interpretation of the results; however, there were consequences to this choice, as tag sites from relatively intact pseudogenes or duplicated genes were left out of the analysis. An illustrative example is provided by *GAPDH *[GeneID:281181], the gene encoding the constitutively expressed glyceraldehyde-3-phosphate dehydrogenase. The tag associated with the 3'-most DpnII site in *GAPDH *was found in seven other locations of the draft genome, making it impossible to infer with certainty whether the tag was generated from *GAPDH *mRNA, especially given that the tag maps within gene boundaries of three other annotated loci in addition to four unannotated, presumably intergenic locations. As a result, this tag associated with *GAPDH *gene expression was not part of the analysis using the Os-fgU subset, and as many as 32.0% of the RefSeq bovine transcripts (16,517 loci with Os-fgU tags versus 24,294 transcripts in RefSeq) were not included in the summary data. This is a problem for all NGS short-read transcriptome approaches, since individual reads from the newer RNAseq methods may also map to multiple places in the genome and may not be unambiguously assigned a single genomic location. This does not preclude closer examination using the comprehensive dataset in the supplementary materials for individual loci to determine whether the BGA data can be used to evaluate expression of confounding tags. In the *GAPDH *example, the other positions in the draft genome to which the tag maps include four apparent pseudogenes where >90% of the *GAPDH *transcript is copied in the draft genome and lacking exons, and another location with intron-carrying similarity to the gene (but lacking upstream exons) and annotated as 'similar to *GAPDH*' (according to GenBank). One might reasonably conclude that all occurrences of the tag are related to *GAPDH *expression and include the tag in analysis; however, such decisions are not practicable to automate on a scale that considers all multiple-mapping tags and are best left for decisions by investigators focusing on specific genes.

A summary of the tissue libraries and characteristics of tags generated from them is found in Additional file 1. The tag data are broken out by tissue, classified according to the BRENDA tissue ontology, and tag-mapping parameters such as number of unique loci mapped, number of unique sense/antisense tag sequences mapping to these loci, abundance of the sense/antisense tags mapping within loci, and mitochondrial genome-encoded expression.

### Bovine tissue classification based on expression profiles

It seems reasonable to expect that similar functions in different tissues will require similar sets of genes to be expressed, such that functional relatedness of tissues is likely to be reflected in shared patterns of transcript abundance. The static transcript profiles created in the BGA reflect the state of the tissues' activity at the time of sampling, and may not always reflect common developmental origin. Therefore, it should be informative to determine how the tissues relate to one another in terms of their expression patterns exemplified by transcript diversity and abundance measures. A straightforward approach is to cluster the tissues based on commonalities in transcript abundance, such as implemented in the Simcluster application [12]. This application was chosen because it was developed and optimized specifically to cluster enumeration (Serial analysis of gene expression (SAGE), massively parallel signature sequencing (MPSS), this BGA data) expression data based on the computed similarity between the transcript tag profiles in a simplex space where the summation of the tag abundances, by definition, is constrained. To put the results in context, the hierarchical Simcluster dendrogram is annotated with the BRENDA anatomical tissue classifications to determine if this classification schema fits with patterns of transcript abundance in cattle tissues.

**Figure 3:**
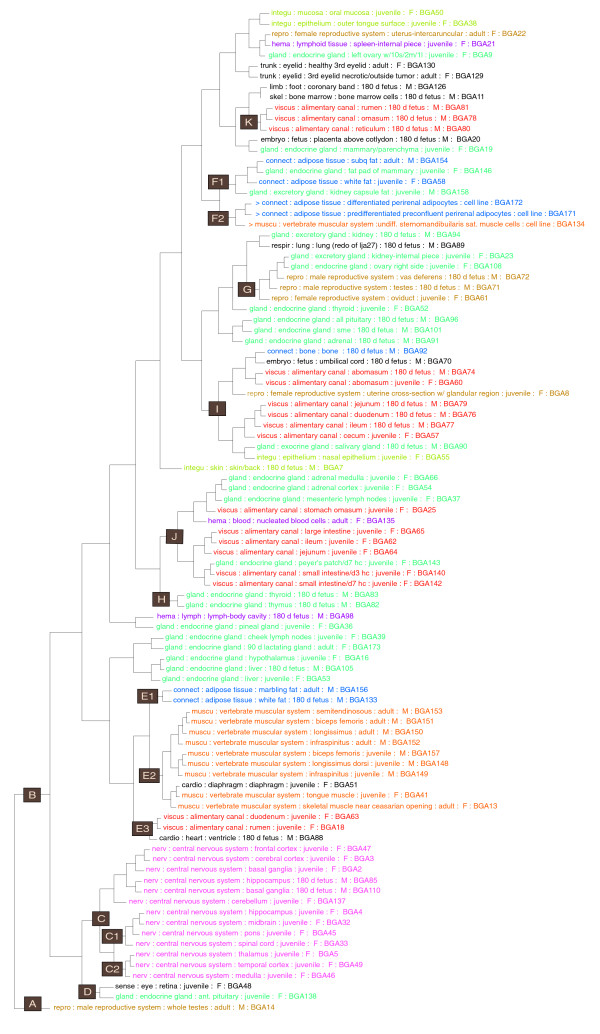
**Simplex clustering (Simcluster) of tissue transcription profiles and their correspondence with BRENDA tissue classification**. Tissue names are colored according to the topmost level of BRENDA tissue classes noted as the first term for each leaf; however, only those classes that had more than three tissue members are given a non-black color.

The hierarchical clustered dendrogram constructed using abundance data from the Os-fgU tag set in Figure 3 illustrates how classification based on BGA data largely reflects the anatomical model at the top-most level of BRENDA ontology. For example, cluster E2 indicated in Figure 3 includes all of the muscles collected from both juvenile and adult animals, and cluster C includes all 13 tissues of the 'nervous tissue' class. These results indicate the validity of using transcript abundance to determine relatedness of tissues. However, not all of the clustering behaves in this fashion; for example, the 'connective tissue' class comprises four adipose samples in the BGA, indicating that adult marbling fat and fetal white fat are closely related to one another and to skeletal muscle in cluster E1, while juvenile white fat and adult subcutaneous (SubQ) fat are substantially different from these two tissues in cluster F1. The analysis of fatty tissues also illustrates a limitation of the ontology system, as clearly the fat pads of the mammary and kidney capsule, which are placed in the gland classification in cluster F1, are more similar to the subcutaneous and juvenile white-fat samples than they are to other members of their own classification. Similarly, the inclusion of the diaphragm, classified as 'cardio', in the muscle cluster E2 is unsurprising, but suggests that diaphragm should also be a child node under skeletal muscle in the BRENDA tissue ontology.

It is interesting to note the clustering of all three cell lines (two adipose cell lines and one satellite cell line) in cluster F2 in Figure 3. The relatively close similarity to several fat tissues (mammary, kidney, juvenile white and adult SubQ) in cluster F1 indicates that the fat cell lines retain transcript profiles approximating their source tissue, but the close relatedness of the muscle satellite cell line suggests that there is a transcriptional profile component common to cell cultures or the satellite cell line has an adipose-related transcript profile. Another interesting result from the clustering is that the adult testis has a tag profile with low similarity to any other tissue, being the sole tissue in cluster A. This presumably reflects that the mature primary sex organ of a mammal has unique sets of gene expression requirements. In contrast, the fetal testis is clustered with fetal vas deferens, juvenile oviduct and ovary, and juvenile kidney in cluster G, presumably because at this immature stage it has not developed the specialized function(s) that distinguish the adult testis.

The expression profile similarity between the juvenile anterior pituitary and retina samples, as indicated in cluster D of Figure 3, is interesting, as a relationship between these tissues is not obvious from an anatomy-based ontology. Some other surprising results include the observation that the three lymph nodes collected (juvenile cheek and mesenteric, fetal body cavity) have relatively distantly related profiles, with cheek being closest to lactating mammary gland, body cavity being closest to the pineal gland, and mesenteric being closest to adrenal medulla and cortex. Similarly surprising is the distant relationship between the fetal and juvenile thyroid samples, with the fetal sample most closely related to fetal thymus and the juvenile less closely related but clustered with the same group of tissues as fetal testis.

Clustering of tissues by expression profile in the alimentary canal is of interest because cattle are ruminants with a more complex digestive system than other mammals. The fetal rumen, omasum, and reticulum, which are compartments of the stomach, are tightly clustered in cluster K, but are distantly related to expression in their juvenile counterparts in clusters J and E3. Similarly, fetal jejunum and ileum sections of small intestine in cluster E3 have similar expression profiles, which are substantially distant from profiles of their juvenile counterparts, probably because of the ongoing digestive processes in the juvenile animal. In contrast, the fetal and juvenile abomasums are clustered in I, perhaps because the secretory functions of this 'fourth stomach' have already begun at 180 days gestation. In terms of the rumen, which has no exact counterpart with other species having broad gene atlas data, the expression profile of the fetal sample in cluster K is closest to those of the fetal samples of coronary band (area above the hoof where hair growth ends) or bone marrow, while the juvenile rumen sample most closely resembles the juvenile duodenum and fetal ventricle patterns in cluster E3.

### Mitochondrial gene expression profiles

**Figure 4:**
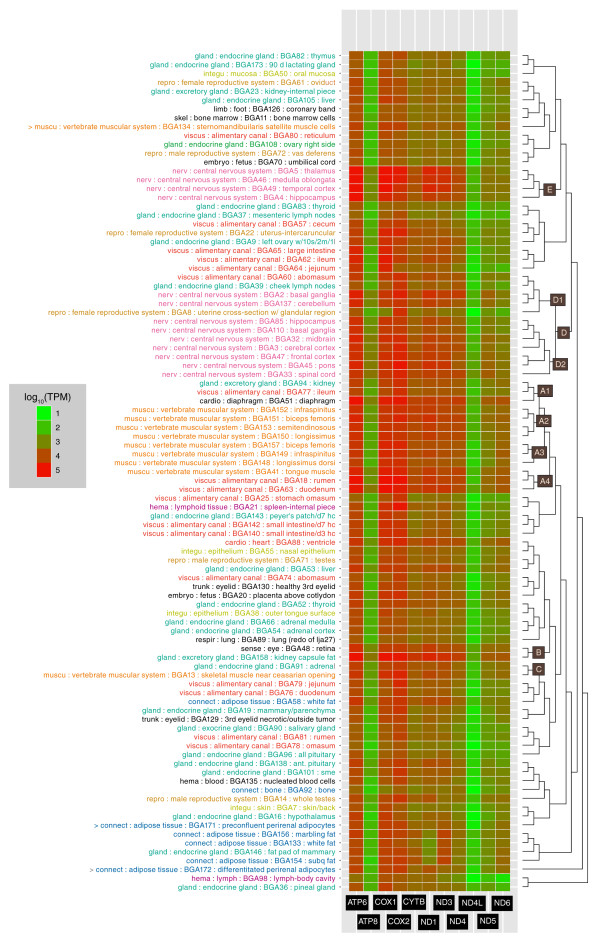
**Simplex clustering (Simcluster) of tissues with mitochondrial tag profiles (TPM) only**. The heatmap shows the absolute abundance of tags associated with each mitochondrial gene. Tissue names are colored according to the topmost level of BRENDA tissue classes noted as the first term for each leaf; however, only those classes that had more than three tissue members are given a non-black color.

The DGE procedure provided data for 9 of the 11 protein-coding genes present in the bovine mitochondrial genome (the *COX3 *and *ND3 *transcripts have no DpnII sites). The data on mitochondrial gene expression were of special interest because of the important role of this organelle in muscle, the most important tissue in beef production; however, these data provided a different perspective on the classification of other tissues as well. A heat map of expression of the nine mitochondrial genes in Figure 4 provides visual context for the basis of clustering used by Simcluster in both of the Simcluster dendrogram (this depiction was not possible for Figure 3 because, instead of the 11 columns in Figure 4, it would require in excess of 200,000 columns). The heat map includes the abundance of all sense and antisense tags mapping within the annotated boundaries of the nine mitochondrial genes, although the contribution of antisense tag abundance is negligible (5%). The heat map illustrates that *ND4L *has the lowest transcript abundance across all tissues, with *ATP6*, *COX1*, and *COX2 *being commonly the most highly abundant. We note that the percentage of antisense tag to total (sense+antisense) tag abundance for the 11 mitochondrial genes is 4.4%. This low percentage indicates that mitochondrial-related tags were generated directionally from mRNA and not from putative contaminating mtDNA, since DpnII sites in mtDNA should have no bias toward sense tag generation.

The relative abundance of mitochondrial genome-derived and nuclear-encoded transcripts was interesting, as in some brain tissues (juvenile thalamus, temporal cortex, and medulla oblongata) the majority of tags observed were derived from these nine mitochondrial genes ( > 57%, range 57.6 to 68.0%; Additional file 1). In contrast, the juvenile hippocampus displayed 34.3% mitochondrial tags and the eight muscle samples averaged 23.6% (range 17.5 to 35.6%). Among all tissues, the average mitochondrial tag abundance was 11.4% of the total of mitochondrial and nuclear-encoded abundance (range 3.5 to 68.0%).

A hierarchical dendrogram of the tissues in the BGA based on mitochondrial tag profiles (right side of Figure 4) shows the clustering of tissues has many similarities to that from the entire set of tags in Figure 3, despite being based on only nine data points per tissue. The skeletal muscles cluster together, with the notable exception that the muscle near the caesarean opening (external abdominal oblique) is less closely related and is clustered with adrenal gland. Also, the tongue muscle clusters with the smooth muscle-containing rumen and duodenum more tightly than with the skeletal muscles, a cluster that would be difficult to predict *a priori*. The fetal rumen and omasum still cluster together, but the fetal reticulum is not a member of the same cluster in the mitochondrial profile, being most closely related to the juvenile ovary. Much of the nervous tissue remains clustered, although the overall clustering is divided into two more distant clusters (D and E in Figure 4), with the principal division related to the much higher tag abundances of three genes (*ATP6*, *COX1*, and *COX2*) in the tissues in cluster E relative to D (see data in Additional file 1). To the best of our knowledge, this is the first time these nervous tissues have been categorized into two distinct groups according to mitochondrial gene expression profiles. Cluster E is especially interesting since this cluster has the highest proportion of mitochondrial to nuclear gene expression and its constituents are the same tissues (medulla oblongata, thalamus, and hippocampus) shown to be enriched in the pathogenic form of the prion protein symptomatic of bovine spongiform encephalopathy [13]. More broadly, mitochondrial dysfunction has been associated with neurological disorders affecting tissues in both clusters D and E [14-16], suggesting that the observed differences in the mitochondrial gene expression profiles may not only be useful in classifying nervous tissue, but also that changes in these differences may provide new insights into the progression of neurological diseases.

Overall, the classification based on all tags shows better agreement with the BRENDA ontology than that based only on nine mitochondrial genes. While this is not surprising, the data on mitochondrial gene expression still provides a new perspective on classification of tissues that are similar in the overall tag profile. For instance, the three cell lines that were clustered together in the full set of tags are quite different in mitochondrial gene expression profile, despite having quite similar percentages of tag abundance derived from the mitochondrial genome (11 to 12%). Moreover, the fact that many tissues are clustered similarly in both profiles supports the existence of a coordination of expression between nuclear and mitochondrial genes.

### Localization of sense and antisense tags within gene models and confounding effects of overlapping genes

The procedure used in creating the BGA should result only in tag sequences reflecting the sequence of expressed, polyadenylated RNA immediately 3' from the DpnII site closest to the polyA tail. Thus, tags mapping to unique genomic locations that lie within gene models, but matching the opposite strand from the predicted mRNA product, represent apparent transcription in the antisense direction (note that tags mapping outside gene models cannot be assigned sense or antisense direction). The fidelity of the tag generation and sequencing process from mRNA is therefore reflected in the propensity of the tags to localize to the DpnII site at the 3' end of the NM or NR RefSeq transcript. The proportion of tags mapping to the 3'-most or next to 3'-most DpnII position on the RefSeq transcripts relative to all tags mapping to the these loci had a mean of 0.906 (0.023 standard deviation (SD)), validating the fidelity of the tag generation and sequencing protocols.

**Figure 5:**
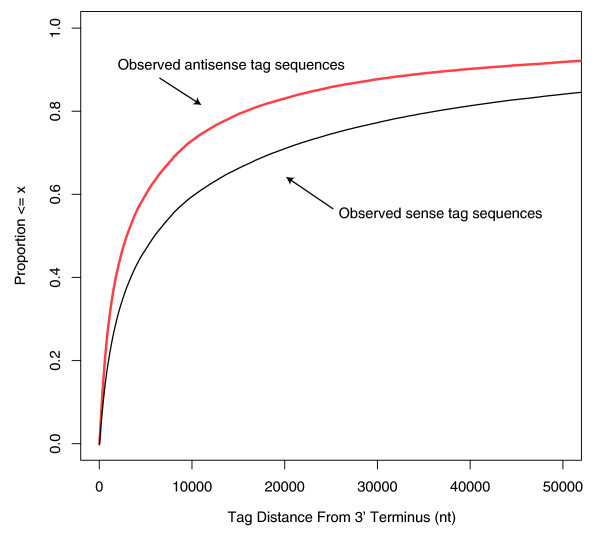
**Empirical cumulative distribution function (ECDF) of the upstream tag sequence distance from the 3' terminus (all distances upstream of the 3' terminus)**. This ECDF plot was created using Os-fgU tags that mapped to all RefSeq transcripts, in either the sense or antisense orientation. Nt, nucleotides.

A comparison of antisense tags to sense tags in all 95 samples in Additional file 1 shows that there were 2.13 times as many observed sense tag sequences versus antisense, while the normalized tag abundance (TPM) of sense tags was 11.5 times that of antisense tags. An analysis of the behavior of the number of unique sense and antisense tag sequences within loci versus the number of unique loci (different GeneID) for every tissue in Additional file 2 shows a looser association of the number of unique sense tag sequences versus unique loci than observed in the antisense case; specifically, when the data were fitted to a quadratic curve, the norm of the residuals in the sense case was 24,208 versus 6,323 TPM in the antisense case, a 3.7-fold difference. A possible explanation for this difference was gleaned from a comparison of the antisense and sense empirical cumulative distribution functions (ECDFs) of the number of tag sequences with respect to their distances upstream of the 3' terminus of the gene model using all tissues in Figure 5. This implies that the only tag sequences accounted for fell within a gene model, while tags mapping outside of annotated gene models were not considered.

**Figure 6:**
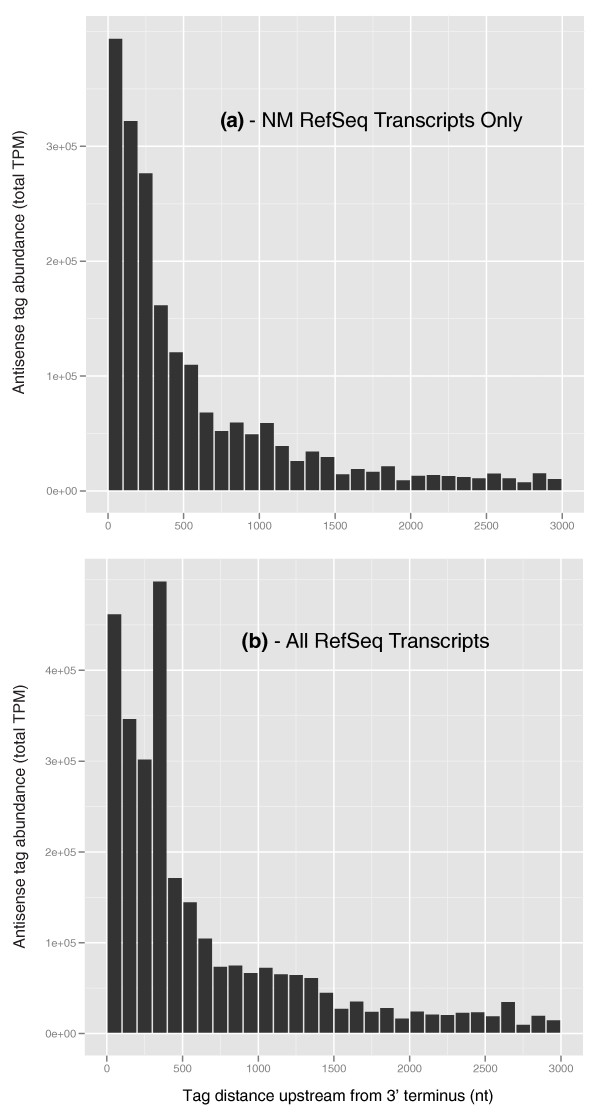
**Comparison of the distributions of antisense tags upstream from the 3' terminus using different RefSeq gene models**. **(a) **Tag abundance-weighted histogram of the upstream distance from the 3' terminus of antisense tag sequences mapping to NM RefSeq transcripts (all distances upstream of the 3' terminus). Os-fgU tags from all libraries were mapped only to NM RefSeq transcripts (curated and considered reliable). **(b) **Tag abundance-weighted histogram of the upstream distance from the 3' terminus of antisense tag sequences mapping to all RefSeq transcripts (all distances upstream of the 3' terminus). Os-fgU tags from all libraries were mapped to both NM and XM RefSeq transcripts. The XM transcripts are model sequences that are considered less reliable than the NM transcripts. Nt, nucleotides.

Figure 5 shows that a larger proportion of the antisense tag sequences are closer to the 3' end of the gene model. This too has been observed in the human 'antisense transcriptome' [17], where antisense transcription was found to be relatively higher in the 1-kb regions upstream (promoter) and downstream (terminator), respectively, of the transcription start and stop sites. The BGA data are consistent with the results of He *et al*. [17], if one takes into account that terminators were much more likely to be observed than promoters because the tags were generated with a heavy 3' bias. These data show that not only were there fewer observed antisense tags than sense tags, the antisense tags tended more towards the 3' terminus than the sense tags, restricting the set of observed tag sequences even further and yielding a closer association of observed antisense tag sequences with number of loci than observed with the sense tags. Precisely quantifying this effect is difficult because of the imprecision of the computational-based gene models, especially with regard to overlapping genes. There were 2,075 tags that were antisense to a gene model in one strand but sense to an overlapping gene model on the complementary strand, accounting for 1,058,662 TPM in all tissues, or 28.5% of the total antisense tag abundance of 3,718,974 TPM from 1,471,248 antisense tag sequences in all tissues. These 2,075 tags constitute a mere 0.141% of all antisense tag sequences. Confidently associating a tag in an overlapping region of the draft genome can be difficult, especially in cases where a tag resides close to the 3' terminus of a gene model, either upstream or downstream. Small changes in these overlapping gene models can have large effects on the relative proportion of antisense to sense total tag abundances by enlarging or contracting the class of 2,075 tags shared by the overlapping gene models. There is evidence that errors are present in gene models associated with antisense tags, suggesting that the class of 2,075 tags shared by the overlapping gene models may change. The tag abundance-weighted histograms of the antisense tag distances upstream of the 3' termini considering only those based on expert reviewed NM transcripts are shown in Figure 6a, while those including all gene models are shown in Figure 6b. The histogram based on NM gene models in Figure 6a exhibits a relatively smoothly decreasing tag abundance-weighted tag sequence count profile as the distance from the 3' terminus increases. This observation, based on thousands of experimentally verified distinct genes, suggests that this profile is reasonably accurate. This profile is different from the one in Figure 6b that includes computationally derived gene models. The inclusion of computationally derived gene models produced a spike in the TPM-weighted tag sequence counts 400 nucleotides upstream of the 3' termini, and is most likely due to errors in the gene models or draft genome assembly. If the tags responsible for this spike at 400 nucleotides can be removed by correcting the likely antisense and sense tag mis-annotation upstream of the 3' terminus, a higher proportion of the antisense tags in Figure 6b would likely shift towards the 3' terminus, increasing the rate at which the antisense curve approaches 1 in the ECDF. Although these corrections will likely significantly affect the overall antisense tag abundance, they will have a much smaller effect on the sense tags because they are 11.5 times more abundant.

### Tissues with atypical transcription tag profiles

Due to the relatively high frequency of incorrectly predicted gene models in the draft bovine genome (especially at the 3' end of transcripts where a high proportion of predicted BGA tags should lie), we used a set of tags that mapped uniquely within the boundaries of genes with expert reviewed, high-quality annotation (Os-fgU tags that mapped to NM/NR in the RefSeq database) to examine general characteristics of tag distributions relative to tissue and tissue class. The tissue with the highest sense tag abundance mapping to the 3' terminus of the NM/NR transcripts is the juvenile female hypothalamus (BGA16) at 499,200 total TPM (49.9% of all tags in this library mapped to NMs and NRs, by definition) compared to all tissues with a mean value of 252,249 TPM (53,104 SD) or 25.2% of all tags mapping to NMs and NRs. Given that this tissue has the highest proportion of tags mapping to the NM/NR RefSeq transcripts, it follows that there should be a lower proportion of tags not corresponding to the 3'-most DpnII site relative to the other tissues - if the tag generation process is working properly. Indeed, the hypothalamus tissue had the second lowest percentage of tags mapping upstream of the 3' end tags at 4.4% relative to all NM/NR tags. The tissue with the lowest percentage was the lactating mammary gland (BGA173) at 2.4%, consistent with its ranking as the third highest percentage of tags mapping to NM/NR transcripts of 37.1%.

The lactating mammary gland is distinctive in at least two other ways. First, this tissue exhibits the lowest percentage (3.18%, range up to 12.7%; Additional file 1) of antisense tag abundance relative to total (antisense + sense tag) abundance for tags mapping within loci, although the juvenile female thalamus (BGA5) at 4.24% and the juvenile female temporal cortex (BGA49) at 5.44% show similar behavior relative to the mean value for all tissues of 10.3% (2.4% SD). Second, the lactating mammary gland exhibits the lowest number of unique GeneIDs with sense tag matches at 8,687 GeneIDs compared to a mean of 11,469 GeneIDs (862 SD), although the distribution of GeneIDs is non-Gaussian (data not shown).

**Figure 7:**
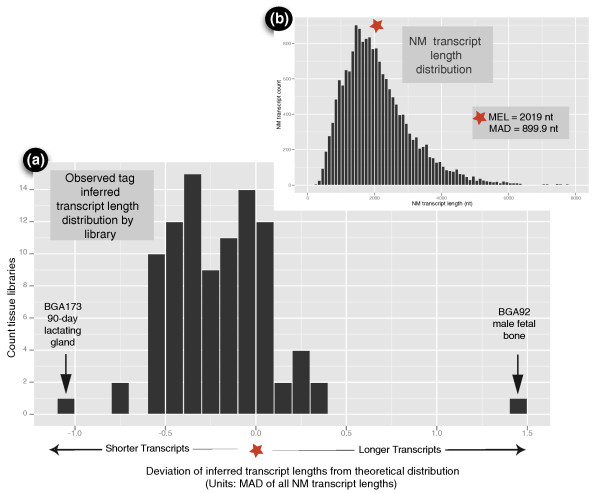
**Distribution of the inferred mean RefSeq transcript lengths by library**. **(a) **Histogram of the tag abundance-weighted mean transcript lengths. The abscissa unit is the mean absolute deviation (MAD) scale estimate (a robust estimate of the dispersion in the data) of all RefSeq NM transcript lengths, while the origin is set to the M-estimator of location (MEL; robust estimate of the mean) of the NM transcript lengths. If the inferred transcript lengths were unbiased, the histogram would be centered on 0 at the red star. The inferred transcript lengths were biased towards shorter transcripts. **(b) **Histogram of the length of all bovine RefSeq NM transcripts. The MEL of this distribution is marked with the red star and corresponds to the origin in (a). Nt, nucleotides.

Characterizing the tissues with independently verifiable predicted physical measures is a powerful mechanism to compare the BGA data with data generated by different experimental methods, such as proteomics. As an illustration, we identified tissues with atypical patterns of tag abundance relative to transcript length for tags mapping uniquely to NM transcripts. First, the extent to which the distribution of inferred NM (protein-coding) transcript lengths of each tissue transcript profile deviates from the distribution of the lengths of all NM transcripts (Figure 7b) was calculated in a manner similar to that used for investigating G+C tag bias [18]. Figure 7a is a histogram of the tag abundance-weighted inferred mean transcript length in each tissue that reveals a modest tag bias towards shorter transcripts, for most libraries, with a MEL (M-estimator of location) of -0.214 (in units of median absolute deviation (MAD) scale estimate of all known NM transcript lengths). Robust estimators of location were used because the distribution in Figure 7a is clearly non-Gaussian. The distribution of inferred transcript lengths in Figure 7a has a MAD scale estimate of 0.2952. Two tissues, BGA173 (90-day lactating gland) and BGA92 (male fetal bone) are outliers at either end of the distribution of inferred transcript lengths. The extreme behavior of these two tissues for the MEL of transcript length was investigated by plotting the inferred transcript length of each NM RefSeq against tag abundance within each tissue.

**Figure 8:**
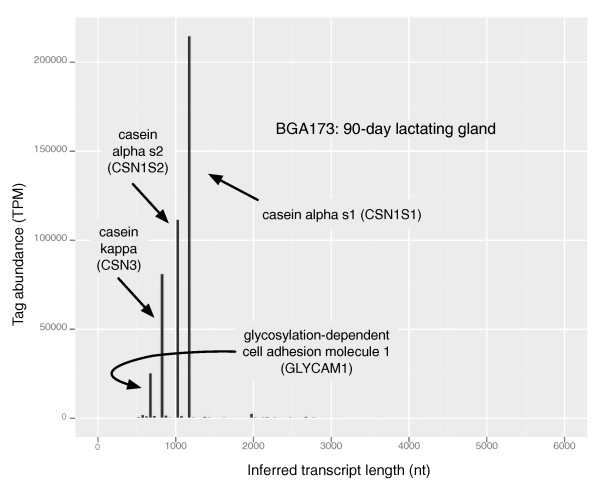
**Histogram of the tag abundance-weighted inferred NM RefSeq transcript lengths for BGA173 (90-day lactating mammary gland)**. The histogram shows the inferred distribution of transcript lengths for the 90-day lactating mammary gland shown in Figure 7a to be composed of atypically shorter transcripts. Nt, nucleotides.

**Figure 9:**
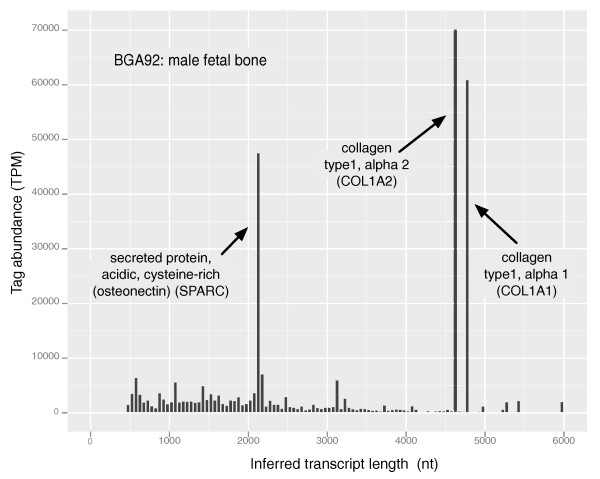
**Histogram of the tag abundance-weighted inferred NM RefSeq transcript lengths for BGA92 (male fetal bone)**. The histogram shows the inferred distribution of transcript lengths for the male fetal bone shown in Figure 7a to be composed of atypically longer transcripts. Nt, nucleotides.

Figures 8 and Figure 9 are histograms of the inferred NM RefSeq tag abundance-weighted transcript length profiles for the juvenile 90-day lactating gland (BGA173) and male fetal bone (BGA92), respectively. In both profiles, tags related to a handful of transcripts dominated the tag abundance. In the case of lactating mammary gland in Figure 8, the tags from just four transcripts/genes dominated. The most abundant transcript was casein alpha s1 (*CSN1S1 *[GeneID:282208]) at 1,172 nucleotides, followed by casein alpha s2 (*CSN1S2 *[GeneID:282209]) at 1,024 nucleotides, casein kappa (*CSN3 *[GeneID:281728]) at 850 nucleotides, then glycosylation-dependent cell adhesion molecule 1 (*GLYCAM1 *[GeneID:282430]) at 679 nucleotides. This result is consistent with previous studies of the lactating mammary gland that indicated these genes were highly expressed [19-21], and demonstrate that the tag abundance-weighted low average transcript length for this library was due to the high expression of a few relatively short transcripts. Although tags were observed that uniquely map to the casein beta (*CSN2 *[GeneID:281099]) genomic region, there are no DpnII sites within the associated RefSeq mRNA [GenBank:NM_181008.2] (98.0% BLAST similarity to the genomic sequence) and, therefore, it is not included in Figure 8. Notably absent in Figure 8 was high expression of the transcript encoding alpha-lactalbumin (*LALBA *[GeneID:281894]), usually abundant during mid-lactation. This was because the tag sequence associated with the 3'-most DpnII site of *LALBA *transcript matches three places in the draft genome sequence, all of which lie near one another on chromosome 5 adjacent to a segment of 144,722 'N' residues (unknown base) and potentially indicating problems with the assembly. Similar to the description of *GAPDH *earlier, the pattern of expression of the tag was consistent with *LALBA *and it was present in high concentration ( > 57,000 TPM) in lactating mammary gland, suggesting that it would be reasonable to assume all instances of the tag were generated from *LALBA*.

The histogram in Figure 9, BGA92 (male fetal bone), exhibits the expected behavior for this 'outlier' tissue. A high number of tags related to two genes with particularly long transcripts, encoding structural collagens, dominated the distribution and led to a high average tag abundance-weighted transcript length. This profile is consistent with actively growing bone tissue and gives a glimpse into which transcripts' abundances dominated at this stage of development.

### Constitutively expressed genes

A set of 'housekeeping' genes was identified from tags that mapped to single genomic locations within the boundaries of genes annotated with NM/NR transcripts (that is, mostly from the Os-fgu-PC set in Figure 2b) and observed in all 95 BGA samples. Variability in the expression level of these genes between tissues was quantified by the coefficient of variation of their tag abundances to identify candidate genes that might be used as normalization controls in cross-tissue gene-expression experiments. A complete list of all 5,429 constitutively expressed genes is presented in Additional file 3, ordered from the genes with the lowest coefficient of variation to the greatest.

A gene's absence from the list in Additional file 3 may be due to a number of factors, including the two discussed earlier: 1) the gene doesn't have a DpnII site, 2) the gene's constitutively expressed tags matches more than one genome location, 3) the gene cannot be mapped to at least one Os-fgU tag in each and every tissue, and 4) that genes previously thought to be constitutively expressed are not in the relatively large number (compared to previous studies) of functional diversity tissues (e.g., coronary band, medulla oblongata, and kidney fat) presented in this study. Consider the case of the canonical housekeeping gene, β-actin (*ACTB*, [GeneID:280979]), predicted to produce a ubiquitously expressed tag that fails to make the list because it matched to multiple places in the draft genome. As with *GAPDH*, another canonical housekeeping gene discussed above, the tag cannot be unambiguously associated with expression of the particular transcript and the BGA data must be carefully interpreted before making conclusions about the variation in expression among tissues. Therefore, the list of constitutively expressed genes should be considered a putative ordering of a list of candidate housekeeping genes with mean values and other statistics validated by other experimental means. However, the data do suggest that the gene encoding stomatin-like 2, *STOML2 *[GeneID:510324], has the lowest coefficient of variation among those in the list and is expressed at a high enough level in all tissues that it may be a potentially useful and stable reference in cross-tissue expression experiments.

### Constitutively expressed and non-genome-mapping tags

The breadth and diversity of the BGA samples were used to identify constitutively expressed genomic regions mapping to the NCBI 4.1 draft genome, mapping both within and outside of the known gene models. There were 5,429 constitutively expressed loci that were associated with 45,178 unique tag sequences, mapping anywhere within the known boundaries of the loci, for a total of 33,261,577 TPM. There were 8,694 unique tag sequences constitutively expressed with a total normalized abundance of 37,290,551 TPM, 5,249 of which were found to map to 4,610 distinct loci (GeneIDs) for a total normalized abundance of 29,402,265 TPM. The remaining 3,445 tags, with a total 7,888,287 TPM, mapped to the draft genome, but outside known gene boundaries. Since these constitutively expressed tags were generated from polyadenylated mRNA, these tags likely correspond to genuine transcripts from incompletely annotated genes. Supporting the proposition that these 3,445 tags originated from extant constitutively expressed transcripts, 2,603 of the 3,445 tags were found to exactly map to contigs *de novo *assembled from normalized bovine adipose tissue transcriptome reads sampled from an animal not contributing to the BGA data (data not shown). Since the library was normalized, it was unlikely that all 3,445 tags would be found in this library.

In a similar analysis, the diversity of the BGA samples was used to identify constitutively expressed tags not mapping to the draft genome. The presence of these tags in all tissues suggests that they are not artifacts, but represent genuine transcripts. There were 206,963 unique tag sequences with an aggregated tag abundance of 9,737,034 TPM (10.8% of Os-F TPM) that did not match the cattle draft genome (Os-fNG in Figure 2b), including the mitochondrial genome. There were 797 tags with 3,022,585 TPM or 31.0% of Os-fNG that did not map to the draft genome but were constitutively expressed, often in relatively high abundance, while those found in fewer libraries were more likely to be found in low abundance. These non-matching tags could be artifacts, genomic regions specific to the individual animals sampled (allelic variants), or they could represent regions of the transcribed genome not included in the draft bovine genome. To check the possibility that some of these non-matching tags were due to single base mismatches, MAQ was used to screen these non-matching tags from positions 5 to 20 (the first four bases representing the DpnII recognition site) against the NCBI 4.1 build of the draft bovine genome and all 1.52 million bovine ESTs in GenBank. Those tags that exhibited single-base mismatches to the draft bovine genome amounted to 139,537 unique tag sequences for 4,938,528 TPM (Os-fng-SMM; Figure 2b) or 50.7% of the non-matching tag abundance (Os-fNG). Mapping the non-matching tags to the bovine ESTs revealed 18,436 Os-fng-EST tags that perfectly matched the ESTs and accounted for 5,542,181 TPM or 56.9% of the non-matching tag abundance (Os-fNG). From the Os-fng-SMM that were a single-base mismatch away from matching a genomic region, 8,767 unique tag sequences with a tag abundance of 2,011,638 TPM (20.7% of the Os-fNG) were also a perfect match to the bovine ESTs. The behavior of the non-matching tags in these different categories, with respect to the number of tissues that they are present in, is shown in Additional file 4.

The constitutively expressed unique non-genome-matching tag sequences at the rightmost position in Additional file 4 are surprisingly few relative to their contribution to the overall non-genome mapping tag abundance. There were 613 tag sequences at 2,693,431 TPM constitutively expressed that mapped to ESTs, amounting to 27.7% of all non-mapping tags. Expanding this list to include constitutively expressed tags with single-base mismatches to the draft genome resulted in 761 tags at 2,970,598 TPM (30.5% of the Os-fNG) that accounted for 95.4% of the constitutively expressed unique tag sequences and 98.3% of the total normalized constitutively expressed tag abundance. These data show that a small fraction (0.37%) of the total number of tag sequences accounts for 31.0% of the total tag abundance of non-genome-matching tags. The observation of constitutively expressed tags suggests that these were derived from real biological entities. While it is difficult to determine the relative contributions of artifacts, genomic variation between individuals, and undiscovered regions of the draft bovine genome, this analysis shows that between 28 and 57% of the total non-matching tag abundance can be associated with observed transcripts, suggesting that the NCBI 4.1 draft bovine genome assembly could be improved to account for these observations. Although the 10.8% of the total tag abundance (Os-F) that does not match the draft genome constitutes a significant fraction of the Os-F, most of the non-matching tags map to previously sequenced transcripts, suggesting that these non-genome mapping tags are not indicative of a systematic problem with tag generation or sequencing.

## Conclusions

The BGA tissue transcriptome resource provides new insights into mammalian biology. Clustering all the tissues based on the similarities of their transcript tag profiles reveals both expected and unexpected associations that, in most cases, confirms the BRENDA ontology and, at least in one case, suggests additional tissue associations. The demonstration of wide variation in the mitochondrial percentage of overall gene expression and the observation of it being relatively extremely high (over 50%) for the juvenile female thalamus, temporal cortex, medulla oblongata, as well as their highly similar mitochondrial transcript profiles, were unexpected. These observations may serve to seed new hypotheses concerning mitochondrial-related neurodegenerative diseases. The lactating mammary gland exhibited the lowest number of expressed distinct genes, the lowest proportion of antisense tags to all tags, and the highest proportion of the shortest transcripts of any of the tissues, reflecting its role as a factory for milk proteins. The distribution of inferred transcript lengths in all tissues is a simple example of computing a metric that can be used to compare the tissues and link the transcriptome profiles with other methods, for instance, proteomic.

Certain antisense and sense tags that both did and did not map to the draft genome were used to show that the NCBI 4.1 draft genome assembly and/or annotation could be improved, most likely in or around computationally derived gene models and overlapping genes. In addition, we provide a list of candidate housekeeping genes and their coefficient of variation that may prove useful in future investigations. The BGA website [8] presents a view of the transcriptome from a genome position perspective, providing a useful resource to address questions concerning the diversity of tissues in which a particular gene, or set of clustered genes, are expressed. This resource will also be useful for studies examining coordinated expression of genes by genomic region.

## Materials and methods

### Tissue collection and total RNA isolation

All bovine tissues were collected following the US Department of Agriculture (USDA) Agricultural Research Service (ARS) animal use and care protocols. Most tissues were snap frozen in liquid N_2 _immediately after excision. Exceptions were bone marrow that was separated from bone with a syringe needle and white blood cells collected as a buffy coat by centrifugation (3000 × g, 4°C, 30 minutes). Residual red blood cells were removed from the buffy coat by resuspending twice in 140 mM NH_4_Cl, 17 mM Tris/HCl and collecting the unlysed cells by repeating centrifugation after each resuspension. All samples were stored at -80°C until sonicated or pulverized and then homogenized using a polytron homogenizer for extraction of total RNA with TRIZOL (Molecular Research Center, Cincinnati, OH, USA), using the manufacturer's protocol. The integrity of the RNA was confirmed by a 2100 Bioanalyzer and RNA 6000 Nano-chip (Agilent, Santa Clara, CA, USA). The samples used had an average RNA integrity number (RIN) value of 8.0 and a 28S:18 S rRNA ratio of 1.42.

### Tag library construction and sequencing

Tag library preparation was performed at Illumina (formerly Solexa, Hayward, CA, USA) using a progenitor version of DGE-Tag Profiling DpnII Sample Prep kit and protocol. In brief, total RNA aliquots (1 or 2 μg) were diluted in 50 ml of nuclease-free H_2_O and heated at 65°C for 5 minutes to disrupt secondary structure prior to incubation with magnetic oligo-dT beads to capture the poly-adenlyated RNA fraction. First- and second-strand cDNA was synthesized and bead-bound cDNA was subsequently digested with DpnII to retain a cDNA fragment from the 3'-most GATC to the poly(A)-tail. Unbound cDNA fragments were washed away prior to ligation with GEX DpnII adapter to the 5' end of the bead-bound digested cDNA fragments. This adapter contains a restriction site for MmeI that cuts 17 bp downstream of the DpnII site. After subsequent digestion with MmeI, 21 bp tags starting with the DpnII recognition sequence were recovered from the beads and dephosphorylated prior to phenol/chloroform extraction. Then, a second adapter (GEX adapter 2) was ligated onto the 3' end of the cDNA tag at the MmeI cleavage site. The adapter-ligated cDNA tags were enriched by a 15-cycle PCR amplification using Phusion polymerase (Finnzymes (now Thermo Fisher Scientific), Lafayette, CO, USA) and primers complementary to the adapter sequences. The resulting fragments were purified by excision from a 6% polyacrylamide Tris-borate-EDTA (TBE) gel. The DNA was eluted from the gel debris with 1× NEBuffer 2 by gentle rotation for 2 hours at room temperature. Gel debris were removed using Spin-X Cellulose Acetate Filter (2 ml, 0.45 μm) and the DNA was precipitated by adding 10 μl of 3 M sodium acetate (pH 5.2) and 325 μl of ethanol (-20°C), followed by centrifugation at 14,000 rpm for 20 minutes. After washing the pellet with 70% ethanol, the DNA was resuspended in 10 μl of 10 mM Tris-HCl, pH8.5 and quantified with a Nanodrop 1000 spectrophotometer. Sequencing using Solexa/Illumina Whole Genome SequencerCluster generation was performed after applying 4 picomoles of each sample to the individual lanes of the Illumina 1G flowcell. After hybridization of the sequencing primer to the single-stranded products, 18 cycles of base incorporation were carried out on the 1G analyzer according to the manufacturer's instructions. Image analysis and base calling were performed using the Illumina Pipeline, where sequence tags were obtained after purity filtering.

### Primers

GEX adapter 1: 5' P-GATCGTCGGACTGTAGAACTCTGAAC; 5' ACAGGTTCAGAGTTCTACAGTCCGAC. GEX adapter 2: 5' CAAGCAGAAGACGGCATACGANN; 5' P-TCGTATGCCGTCTTCTGCTTG. GEX PCR primer 1: 5' CAAGCAGAAGACGGCATACGA. GEX PCR primer 2: 5' AATGATACGGCGACCACCGACAGGTTCAGAGTTCTACAGTCCGA. GEX sequencing primer: 5' CGACAGGTTCAGAGTTCTACAGTCCGACGATC.

### Tag processing

The sequence, abundance, and position in the draft genome of each of the tags were stored within the database backends of GBrowse [22] and Identitag [23]. Representing the tags within the GBrowse database was a convenient way to visualize the tags within a gene-centric context, while the Identitag data were optimized for tag-centric queries. The tag sequences were provided in fastq files for each library. Each fastq library file was processed with a custom perl script that aggregated identical sequences and produced a tag library file with three columns: unique sequence, raw tag count, and normalized tag count in TPM. Each library was normalized to contain 1 million TPM. The tag library files were processed though a customized version of the Identitag pipeline that created and populated a MySQL database of tags for all libraries. The adaptor sequence GTCGGACTGTAGAACT constituted 4,047,333 total raw tag counts and 1,235,500 total normalized tag counts (TPM). Tags with the adaptor sequence or indeterminate bases were filtered out for subsequent analyses.

### Tags mapping to the draft genome

The tags in the Identitag database were related to bovine genomic position and annotation by processing the tags in the Identitag database with a custom perl script. The purpose of this perl script was two-fold: to create tag-mapping files that could be imported into GBrowse; and to populate two tables within the Identitag database that linked the tags mapping within gene boundaries in the Identitag database to the loci annotated in the GBrowse database. Tag-mapping genome coordinates, tag, and library information were written to GFF3 files with this script. These GFF3 data were subsequently parsed into the database using the standard GBrowse parsing tool, bp_fast_load_gff.pl. The script used regular expression searching of the bovine genomic (NCBI version 4.1) contig FASTA files for all possible 20-base tags beginning with the DpnII restriction site, GATC, resident on either strand of the genomic DNA. All putative tags discovered were queried for their presence in the Identitag database, while the tags' locations were determined using the fdata table in the GBrowse database. If a tag was present within a gene boundary and in the Identitag database, the script associated and recorded the gene's unique GBrowse identifier (fdata.fid) with the tag's unique Identitag identifier in a dedicated table. Because a tag may be present in more than one tissue, this association was performed for all tag-tissue combinations. This script also associated the gene's unique GBrowse identifier with the tag sequence, the tag's starting and ending positions, and strand on the genomic contig in a separate table.

### Computed tag characteristics

Often used computed tag characteristics such as tag sequences passing the significance filter (tag abundance greater than 2 TPM in at least one library or present in at least ten libraries) were computed with SQL queries of the Identitag and GBrowse databases and parsed into their own tables in the Identitag database. Typical examples include the number of times an observed tag sequence was found to match the draft genome, the number of tags for each locus (GeneID), constitutively expressed genes, and tags not matching the draft genome. The R computing environment [24] was used to calculate the empirical cumulative distribution functions and histograms using the heR package [25], robust estimators were calculated with the Rallfun-v7 functions [26], and most plots were created using the ggplot2 package [27]. MAQ [28] (version 0.7.1) was used with default parameters to map the non-genome mapping tags to the draft genome allowing for a single base mismatch between tag base positions 5 to 20. MAQ was also used to find all non-genome mapping tags that perfectly map any *Bos taurus *EST in GenBank. Simcluster [12] was used to plot the similarities in the transcriptional tag profiles with complete linkage.

### Data availability

The data are archived at the NCBI Gene Expression Omnibus (GEO) under accession [GEO:GSE21544] [29].

## Abbreviations

BGA: Bovine Gene Atlas; bp: base pair; BRENDA: Braunschweig Enzyme Database; DGE: digital gene expression; ECDF: empirical cumulative distribution function; EST: expressed sequence tag; MAD: median absolute deviation; MEL: M-estimator of location; NGS: next generation sequencing; Os: Operative set of tags where all bases were defined; Os-F: subset of Os that passes the 2 TPM or ten library filter; Os-fG: subset of Os-f that maps to the draft bovine genome; Os-fgU: subset of Os-fG that maps uniquely to draft bovine genome; Os-fgu-PC: subset of Os-fgU that map to protein coding genes; Os-fNG: subset of Os-f that does not map to the draft genome; Os-fng-EST: subset of Os-fNG that matches any of the 1.52 × 10^6 ^bovine EST in GenBank; Os-fng-SMM: subset of Os-fNG that matches the draft genome allowing for a single base mismatch from tag base 5 to 20; Os-G: subset of Os that maps to draft bovine genome; RIN: RNA integrity number; SD: standard deviation; SubQ: subcutaneous; TBE: Tris-borate-EDTA; TPM: tags per million; UTR: untranslated regions.

## Authors' contributions

GPH developed the data processing pipelines, website, and database schema. GPH, TSS, TPLS, and LJA developed the concept of the BGA resource and wrote the manuscript. LJA collected the Hereford tissues and extracted total RNA. TSS standardized RNA samples for cDNA library construction. CDH supervised quality control of all incoming RNA, library preparation, sequence production, and all outgoing datasets. GPH, JWK, TSS, LKM, SMS, and CPVT analyzed the data. CRG, SMB and SCB adapted the BGA database and host the BGA website.

## Supplementary Material

Additional file 1 **Tissues sampled and annotation**. BRENDA tissue ontology terms are given when known, with the hyphenated number at the end of the column headings specifying level of granularity, where 1 is the lowest level of granularity and the broadest scope. Only tags mapping uniquely to the draft genome that passed our filters (Os-fgU; Figure 2b) were considered. Total Sense TPM Within NM & NR Transcripts: sum of normalized tag counts (tags per million) that mapped on the same strand and within the boundaries of NM and NR transcripts. On 3' terminus: sum of normalized tag abundance of those tags that mapped on the same strand and at the 3'-most and next to 3'-most DpnII sites on the NM or NR transcript (Figure 2a). Not on 3' terminus: sum of normalized tag abundance of those tags that did not map to the 3'-most and next to 3'-most DpnII sites on the NM or NR transcript (Figure 2a): Count Sense GeneID: total number of unique GeneIDs associated with the loci that the tags mapped to on the same strand and within the boundaries of the annotated genes. Count Sense Tags within Loci: number of unique tag sequences that mapped within the boundaries of annotated genes. Total Sense TPM within Loci: sum of normalized tag abundance of those tags that mapped on the same strand and within the boundaries of all annotated genes. The headings including the word 'AntiSense' are similarly defined, except that these tags mapped to the strand opposite to that of the annotated gene. Click here for file

Additional file 2 **(a) **Association of the number of unique sense tag sequences within protein-coding loci with the number of unique protein coding loci for each tissue library. CSTL_PC: count unique sense tag sequence within protein-coding loci. CSG_PC: count sense protein-coding loci from Additional file 1. Each data point represents a single tissue library. The top pane shows that number of sense tags found with protein coding loci varies quadratically with the number of protein coding loci. The bottom pane shows the residuals from the fit of the data to the quadratic above. **(b) **Association of the number of unique antisense tag sequences within loci with the number of unique loci for each tissue library. CATL_PC: count unique antisense tag sequence within protein-coding loci. CAG_PC: count antisense protein-coding loci from Additional file 1. Each data point represents a single tissue library. The top pane shows that number of antisense tags found with protein coding loci varies quadratically with the number of protein coding loci. The bottom pane shows the residuals from the fit of the data to the quadratic above. Click here for file

Additional file 3 **Housekeeping genes**. Loci that were observed to be ubiquitously expressed were ordered according to the coefficient of variation in the sum of the observed normalized tag abundance (TPM) of all tags mapping on the same strand and within the boundaries of the gene. Only tags mapping uniquely to the draft genome that passed our filters (Os-fgU; Figure 2b) were considered. Click here for file

Additional file 4 **Classification of non-genome mapping tags with respect to the number of libraries they were present in and their mapping behavior with respect to EST and genome mismatches**. This figure shows that the dominant class of non-genome mapping tags are those that are found in nearly every tissue and that most of these map to ESTs or exhibit a single base mismatch from genome mapping tags. Click here for file
