# Supplementary figures and images for: An atlas of bovine gene expression reveals novel distinctive tissue characteristics and evidence for improving genome annotation
Source: Genome Biol. 2010 Oct 20;11(10):R102. doi: 10.1186/gb-2010-11-10-r102 (PMC3218658; doi:10.1186/gb-2010-11-10-r102)

# A - Sense Tags

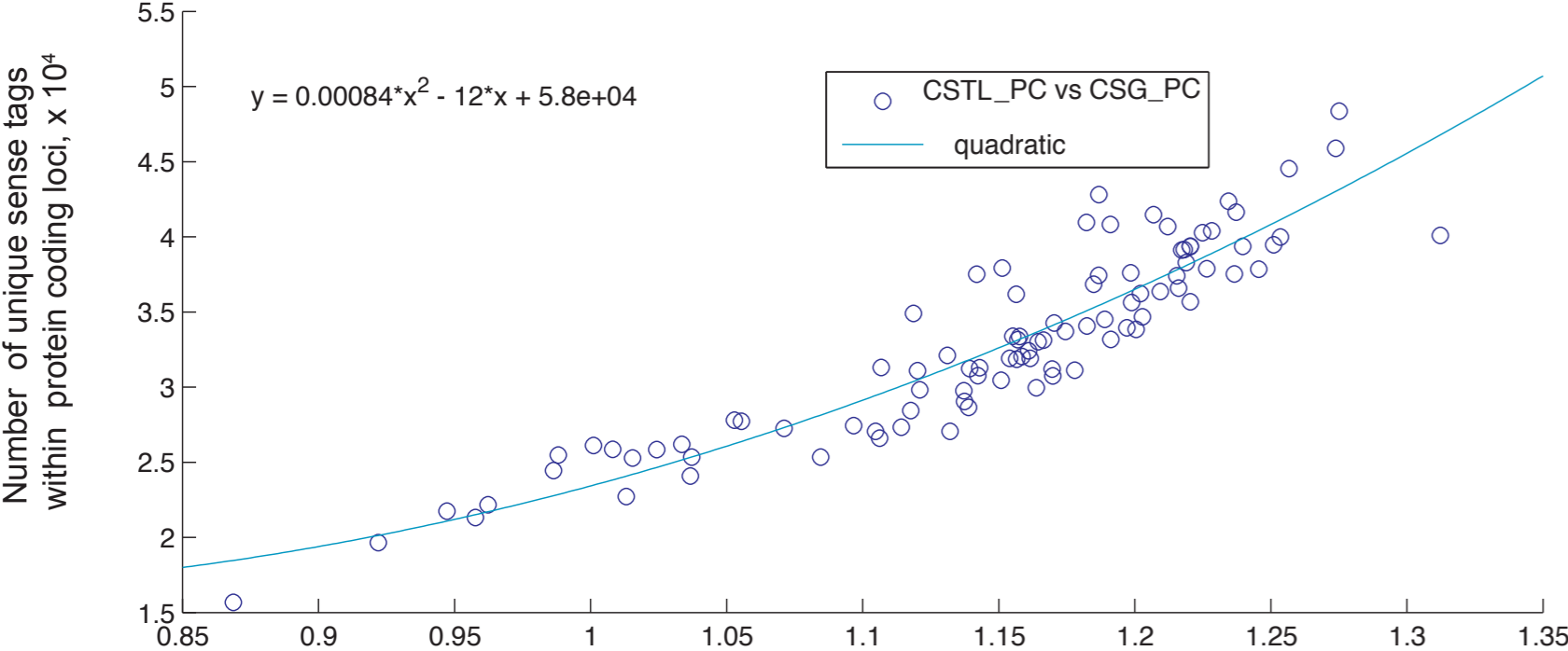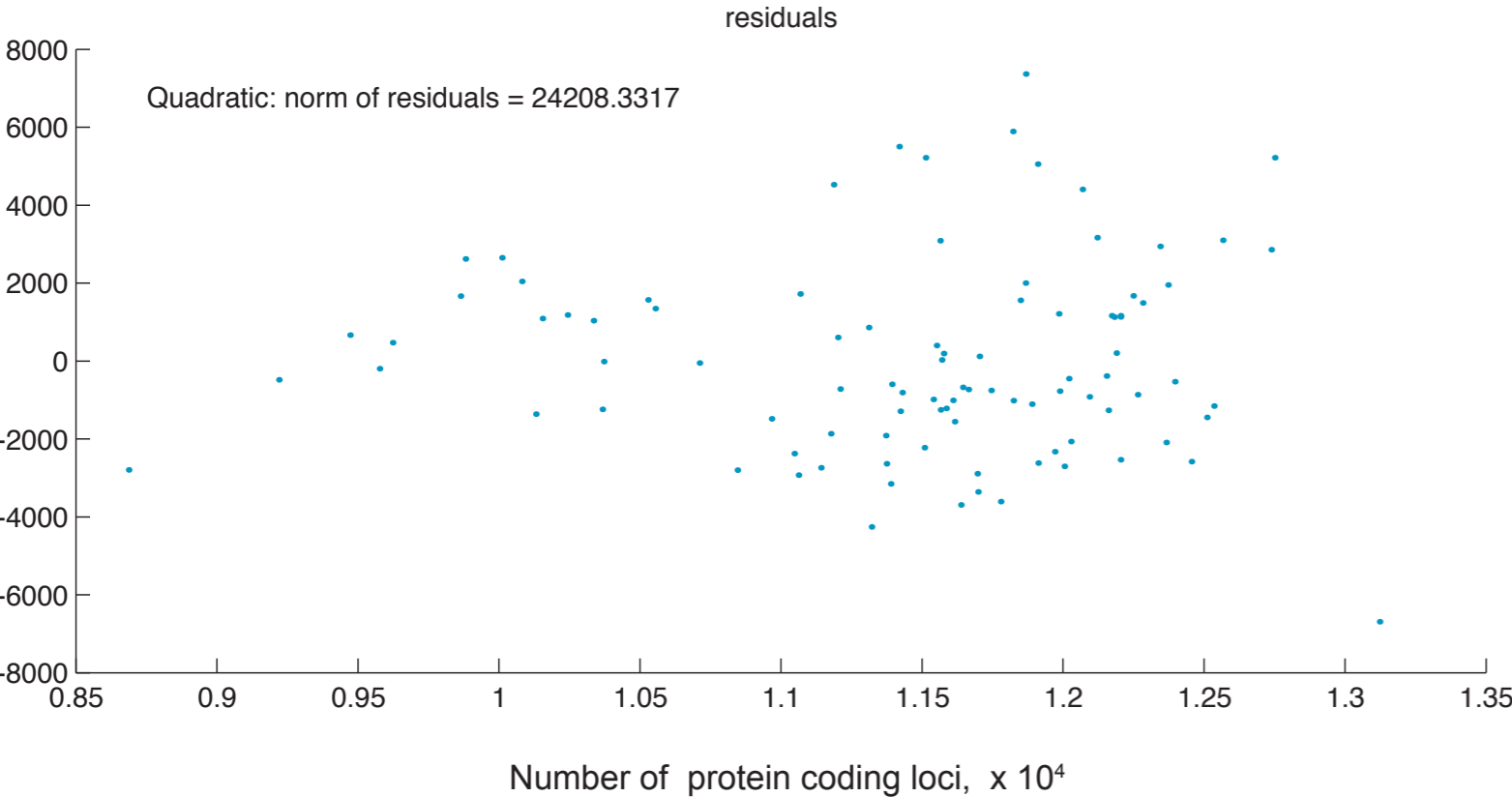

# B - Antisense Tags

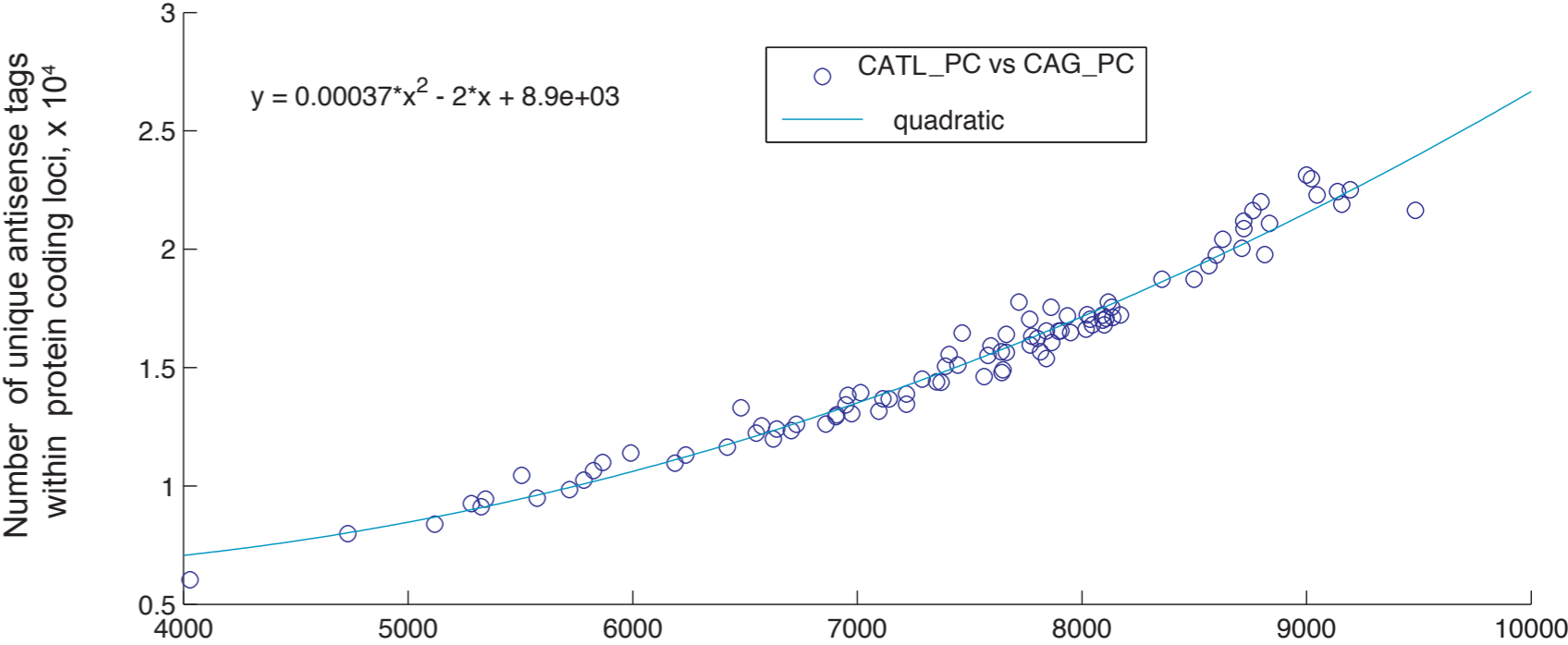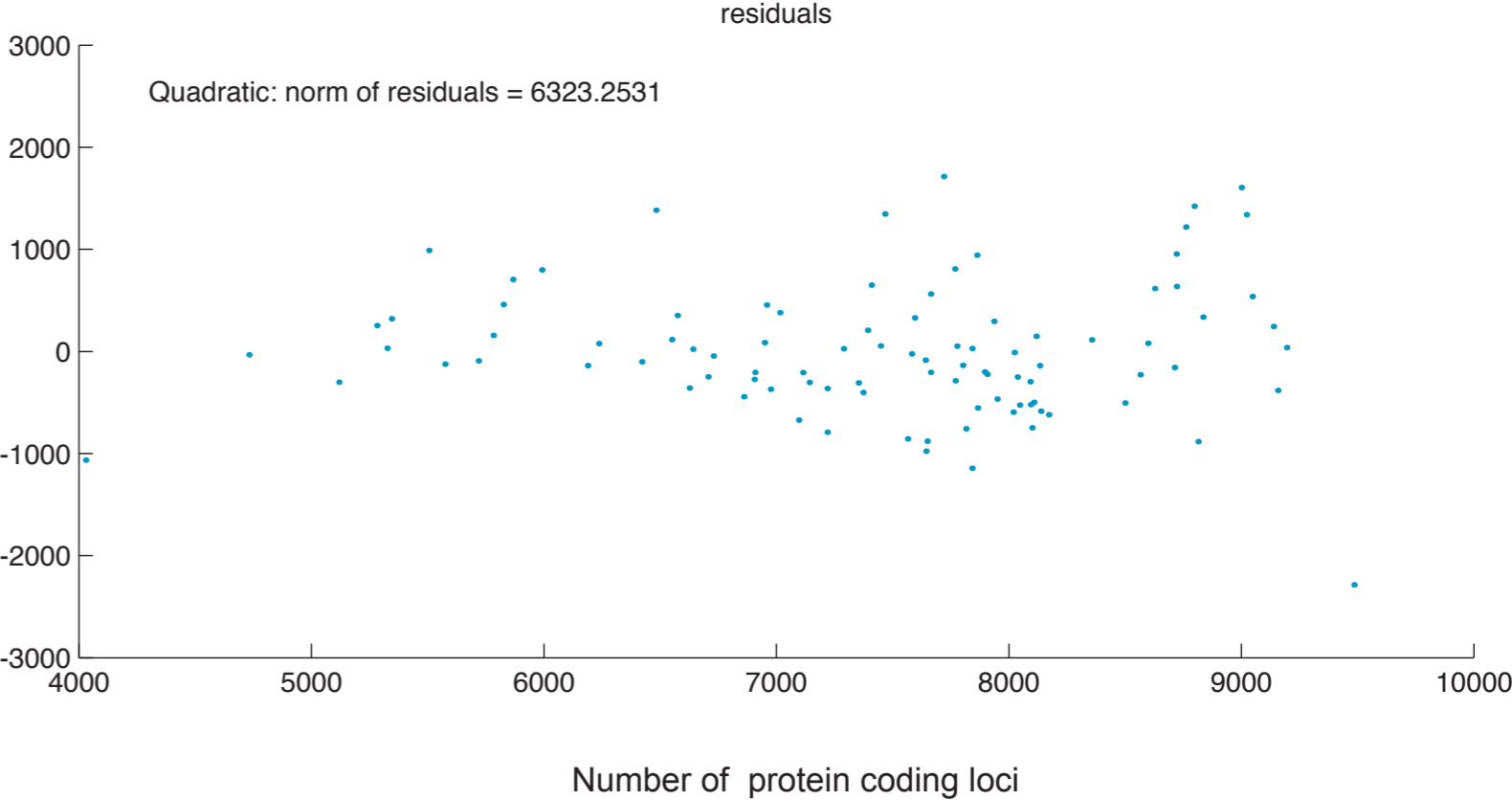

Supplement: Additional file 2 — (a) Association of the number of unique sense tag sequences within protein-coding loci with the number of unique protein coding loci for each tissue library. CSTL_PC: count unique sense tag sequence within protein-coding loci. CSG_PC: count sense protein-coding loci from Additional file 1. Each data point represents a single tissue library. The top pane shows that number of sense tags found with protein coding loci varies quadratically with the number of protein coding loci. The bottom pane shows the residuals from the fit of the data to the quadratic above. (b) Association of the number of unique antisense tag sequences within loci with the number of unique loci for each tissue library. CATL_PC: count unique antisense tag sequence within protein-coding loci. CAG_PC: count antisense protein-coding loci from Additional file 1. Each data point represents a single tissue library. The top pane shows that number of antisense tags found with protein coding loci varies quadratically with the number of protein coding loci. The bottom pane shows the residuals from the fit of the data to the quadratic above. [file gb-2010-11-10-r102-S2.PDF]

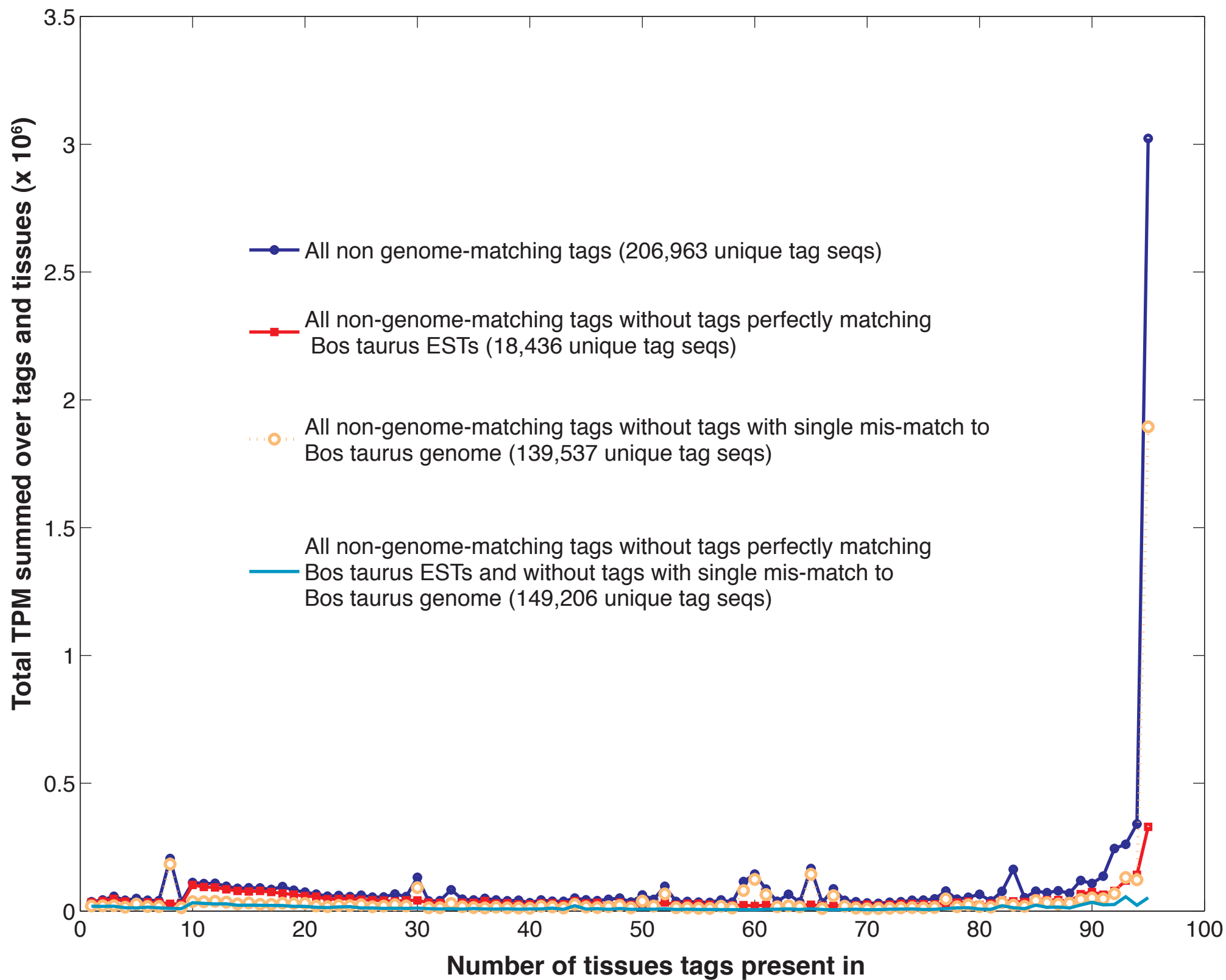

Supplement: Additional file 4 — Classification of non-genome mapping tags with respect to the number of libraries they were present in and their mapping behavior with respect to EST and genome mismatches. This figure shows that the dominant class of non-genome mapping tags are those that are found in nearly every tissue and that most of these map to ESTs or exhibit a single base mismatch from genome mapping tags. [file gb-2010-11-10-r102-S4.PDF]
